# Relationship of Oct-4 to malignant stage: a meta-analysis based on 502 positive/high Oct-4 cases and 522 negative/low case-free controls

**DOI:** 10.18632/oncotarget.5737

**Published:** 2015-11-09

**Authors:** Beilong Zhong, Yan Lin, Yingrong Lai, Fangfang Zheng, Xiaobin Zheng, Rijiao Huang, Weilin Yang, Zhenguang Chen

**Affiliations:** ^1^ Department of Thoracic Surgery, The Fifth Affiliated Hospital, Sun Yat-sen University, Zhuhai, Guangdong 519000, China; ^2^ Department of Breast Disease, Peking Union Medical College Hospital, Peking Union Medical College, Beijing 100730, China; ^3^ Department of Pathology, The First Affiliated Hospital, Sun Yat-sen University, Guangzhou, Guangdong 510080, China; ^4^ Department of Pediatrics, The Fifth Affiliated Hospital, Sun Yat-sen University, Zhuhai, Guangdong 519000, China; ^5^ Department of Respiratory Medicine, The Fifth Affiliated Hospital, Sun Yat-sen University, Zhuhai, Guangdong 519000, China; ^6^ Department of Clinical Laboratory, The Fifth Affiliated Hospital, Sun Yat-sen University, Zhuhai, Guangdong 519000, China; ^7^ Department of Cardiothoracic Surgery of East Division, the First Affiliated Hospital, Sun Yat-sen University, Guangzhou, Guangdong 510080, China; ^8^ Department of Thoracic Surgery, The First Affiliated Hospital, Sun Yat-sen University, Guangzhou, Guangdong 510080, China; ^9^ Lung Cancer Research Center of Sun Yat-sen University, Guangzhou, Guangdong 510080, China

**Keywords:** Oct-4, cancer staging, TNM staging, cancer grade of differentiation, meta-analysis

## Abstract

**Background:**

Octamer 4 (Oct-4), an important member of the POU domain transcription factor family, has been suggested to function as a master switch during differentiation of human somatic cells and more recently has come to be linked with neoplastic properties. The aim of this study was to evaluate the relationship between Oct-4 and cancer stage using a meta-analysis approach.

**Materials and Methods:**

Relevant articles published as of May 2015 were retrieved from the following databases: PubMed, ISI Web of Knowledge, Embase, and Chinese National Knowledge Infrastructure (CNKI). The strengths of relationship for outcomes of interest were estimated based on odds ratios (ORs) and 95% confidence intervals (CIs).

**Results:**

A total of 11 articles on Oct-4 and cancer staging that collectively included 502 positive/high Oct-4 cases and 522 negative/low case-free controls were chosen. Positive/high Oct-4 was significantly associated with cancer stage in several kinds of cancer. Specifically, positive/high Oct-4 was associated with cancer stage III/IV (fixed effects: OR = 1.53, 95% CI = 1.12–2.10), primary tumor (T_3–4_) (random effects: OR = 1.93, 95% CI = 0.99–3.77), and cancer grade of differentiation (intermediate-poor) (random effects: OR = 3.45, 95% CI = 1.5–7.61).

**Conclusion:**

These findings suggest that positive/high Oct-4 is more strongly linked to stage III/IV cancer and cancer grade of differentiation, and is correlated with malignant characteristics that lead to poor prognosis in different types of cancer, especially in Asian. Given variability related to ethnicity and differences in cancer types, additional studies are warranted to establish the generalizability of our findings.

## INTRODUCTION

Expression signature of the stemness state of primary tumors may represent a specific approach for identifying patients who are most likely to suffer recurrence or develop metastases. [1] Actually the overexpression of several genes regulating stem cell properties has been documented in cancer tissues suggesting a possible prognostic role. For example, positive/high nestin may be more strongly linked to advanced cancer stage and correlated with malignant characteristics that lead to poor prognosis in different cancers, especially lung cancer. [2] Aldehyde dehydrogenase 1 activity marked breast cancer cells enriched for stem cell properties, at the same time, a prognostic role of SOX2 seemed to be a more suitable marker of early recurrence in breast cancer. [1, 3] Other data suggested that increased level of ZFP57 and decreased expression of CPT1A and CPT1C were associated with high grade glioblastoma. [4] On the other hand, Octamer 4 (Oct-4), a member of the POU-domain transcription factor family, is normally expressed in both adult and embryonic stem cells, [5, 6] but it has also been found to be expressed in both mouse and primordial germ cells. [7, 8] Extensive investigations have revealed that Oct-4 is expressed in some cancer cell types, such as breast, prostate, hypopharyngeal, bladder, lung, esophageal, and hepatocellular cancer. [9–15]

Recent reports have demonstrated that Oct-4 is not only involved in controlling the maintenance of stem cell pluripotency, it is also responsible for the unlimited proliferative potential of stem cells, suggesting that Oct-4 serves as a master switch during differentiation of human somatic cells. [16–18] Moreover, overexpression of Oct-4 increases the malignant potential of tumors, and downregulation of Oct-4 in tumor cells inhibits tumor growth, suggesting that Oct-4 might contribute on maintaining the survival of cancer cells. [19, 20] Some characteristics of Oct-4 seemed involve as a functional switch related to the “stemness” of the cancer or cancer stem cells, which had specific ability to give rise to all cell types found in a particular cancer sample, and persist in tumors as a distinct population and cause metastasis by giving rise to new tumors.

Some subsequent studies of Oct-4 have also reported a link between Oct-4 and malignant characteristics, and suggested that abundant Oct-4 expression correlates with greater malignancy and poor prognosis in different cancer types. To date, however, there has not been an affirmative conclusion about the association of Oct-4 with malignancy. Thus, clarifying the contribution of Oct-4 to malignant properties requires an evaluation of the relationship between Oct-4 and cancer stage.

Although several studies have addressed this relationship, including Zhang et al., [21] Ge et al., [22] Dong et al., [23] Chen et al., [24] He et al., [25] Zhang et al., [26] Li et al., [27] Takako et al. [28] and Ji et al., [29] only some reported a strong association between positive/high Oct-4 and cancer stage. Because the limited sample size of individual studies limits their statistical power, it is important to summarize otherwise inconclusive results across multiple studies to provide evidence for an association of positive/high Oct-4 with cancer stage.

Cancer stage is determined using the TNM (primary tumor, lymph nodes, and distant metastasis) classification system, which has been in worldwide use across all medical specialties for five decades. [30] The TNM system is based on the anatomic extent of the tumor as determined clinically and, in most instances, histopathologically. [30, 31] TNM Staging Standard is a regularly updated manual for the classification of malignant tumors, published by the American Joint Committee on Cancer (AJCC) and the Union for International Cancer Control (UICC) for Malignant Tumors (4^th^ edition). Currently, TNM staging is widely used throughout the world because of its simplicity and prognostic ability. In oncology, TNM staging can facilitate diagnosis, prognosis, treatment, and other clinical decisions. With the 7^th^ edition of AJCC on the National Comprehensive Cancer Network (NCCN) guidelines of esophageal cancer, cancer grade of differentiation was added to the TNM staging system for the evaluation of esophageal cancer stage.

Meta-analysis is a statistical technique for combining results from different studies to produce a single estimate of the major effect with enhanced precision. [32] In order to elucidate the association of positive/high Oct-4 with cancer stage, we carried out a meta-analysis of all eligible studies. Furthermore, we guided subgroup analyses by stratification according to the primary tumor and cancer grade of differentiation.

## RESULTS

### Study characteristics

**Figure 1:**
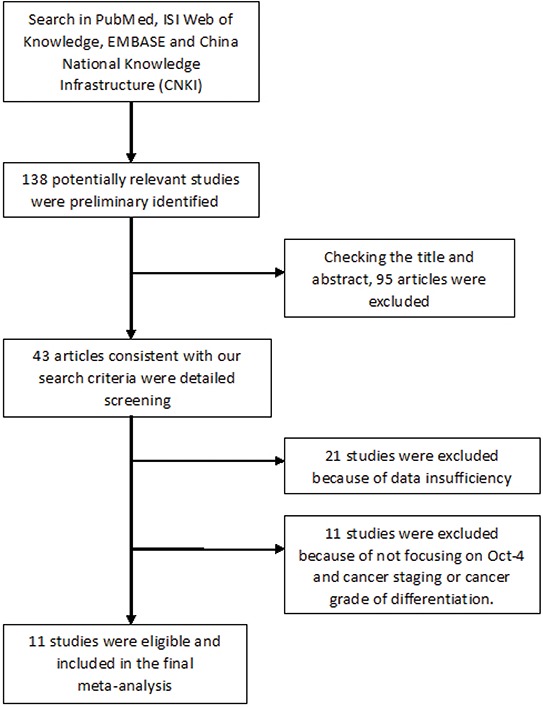
Flow chart of study selection

Our initial search strategy identified 138 potentially relevant studies. After reviewing the title and abstract, a total of 43 articles consistent with our search criteria were preliminarily chosen for further detailed evaluation. After careful screening, 21 studies were excluded because the data were insufficient for our analysis. Of these 21 studies, 11 did not focus on Oct-4 and cancer staging. Ultimately, 11 studies on Oct-4 and cancer staging were deemed eligible for the final analysis; collectively, these studies included a total of 502 positive/high Oct-4 cases and 522 negative/low case-free controls. The characteristics of the included studies are listed in Table 1. All studies were related to clinical research, including three lung cancer studies, two esophageal squamous cell carcinoma studies, one hypopharyngeal squamous cell carcinoma study, one hepatocellular cancer study, one head and neck squamous cell carcinoma study, one ovarian cancer study, one breast cancer study, and one cervical squamous cell cancer study. All cancers were confirmed pathologically. The study selection process is shown in Figure 1.

**Table 1:** Characteristics of the studies included in the meta-analysis

First author	Year	Country	Ethnicity	Cancer type	Total number of patients	Median age (years)
Zhang	2010	China	Asian	LA	112	57
Ge	2010	China	Asian	HSCC	85	60
Chen	2012	China	Asian	Lung cancer	113	57.2
Dong	2012	China	Asian	Hepatocellular cancer	152	NR
He	2012	China	Asian	ESCC	153	56.4
Lilly	2012	America	Caucasian	HNSCC	110	NR
Li	2012	China	Asian	ESCC	50	62
Zhang	2013	China	Asian	Ovarian cancer	74	NR
Li	2013	China	Asian	Lung cancer	102	NR
Ji	2014	China	Asian	CSCC	43	49.6
Takako	2014	Japan	Asian	Breast cancer	93	NR

Abbreviations: CSCC, cervical squamous cell cancer; ESCC, esophageal squamous cell carcinoma; HSCC, hypopharyngeal squamous cell carcinoma; HNSCC, head and neck squamous cell carcinoma; LA, lung adenocarcinoma; NR, not reported.

### Publication bias

**Figure 2:**
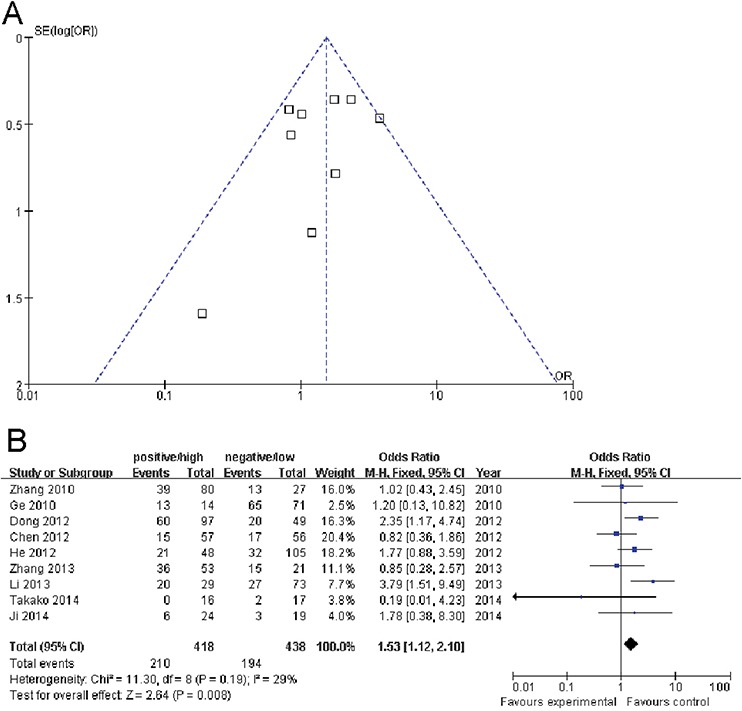
**A.** Funnel plot assessment of evidence for publication bias from nine studies (positive/high Oct-4 vs. negative/low Oct-4 in cancer staging). **B.** Forest plots of Oct-4 and cancer stage III/IV in all cases (positive/high Oct-4 vs. negative/low Oct-4). The squares and horizontal lines correspond to the study-specific OR and 95% CI. The area of the squares reflects the weight (inverse of the variance). The diamond represents the summary OR and 95% CI.

Because part of the information was not complete in two studies, a funnel plot analysis was performed from nine studies to assess publication bias (Figure 2A). It showed no obvious asymmetry in any cancer staging parameter (positive/high Oct-4 versus negative/low Oct-4), and the results revealed no publication bias (*P* > 0.05).

### Meta-analysis results

TNM staging data relating to primary tumor, lymph node involvement and cancer grade of differentiation were extracted. The frequency of primary tumor, lymph node involvement, cancer grade of differentiation and cancer stage in positive/high and negative/low Oct-4 groups are shown in detail in Tables 2 and 3.

**Table 2:** Positive/high Oct-4 and negative/low Oct-4 frequency in cases and controls: TNM stage

FA	Years	Cases (positive/high)							Controls (negative/low)						
		Total	T_1–2_	T_3–4_	N_0_	N_1–2_	S_1_	S_2_	Total	T_1–2_	T_3–4_	N_0_	N_1–2_	S_1_	S_2_
Zhang	2010	80	NR	NR	NR	NR	41	39	27	NR	NR	NR	NR	14	13
Ge	2010	14	3	11	7	7	1	13	71	18	53	59	12	6	65
Chen	2012	57	35	22	24	33	42	15	56	43	13	22	34	39	17
Dong	2012	103/97	31	72	NR	NR	37	60	48/49	13	35	NR	NR	29	20
He	2012	48	13	35	27	21	27	21	105	31	74	72	33	73	32
Lilly	2012	46	6	40	NR	NR	NR	NR	64	38	26	NR	NR	NR	NR
Li	2012	31	8	23	8	23	NR	NR	19	8	11	5	14	NR	NR
Zhang	2013	44/53	NR	NR	24	20	17	36	18/21	NR	NR	7	11	6	15
Li	2013	29	NR	NR	NR	NR	9	20	73	NR	NR	NR	NR	46	27
Ji	2014	19/24	NR	NR	16	3	18	6	15/19	NR	NR	9	6	16	3
Takako	2014	17/16	16	1	11	6	16	0	18/17	18	0	8	10	15	2

Abbreviations: FA, first author; T, tumor; N, lymph nodes; S_1_, stage I/II; S_2_, stage III/IV; NR, not reported.

**Table 3:** Positive/high Oct-4 and negative/low Oct-4 frequency in cases and controls: grade of differentiation

FA	Year	Cases (positive/high)				Control (negative/low)			
		Total	Well	Moderate	Poor	Total	Well	Moderate	Poor
Zhang	2010	80	19	49	12	27	4	13	10
Ge	2010	14	5	8	1	71	29	31	11
Chen	2012	57	3	14	40	56	24	20	12
Dong	2012	NR	NR	NR	NR	NR	NR	NR	NR
He	2012	48	5	12	31	105	48	48	9
Lilly	2012	NR	NR	NR	NR	NR	NR	NR	NR
Li	2012	31	6	21	4	19	8	10	1
Zhang	2013	53	13	19	21	21	13	5	3
Li	2013	29	4	NR	NR	73	34	NR	NR
Ji	2014	24	NR	NR	11	19	NR	NR	9
Takako	2014	17	NR	NR	7	18	NR	NR	3

Abbreviations: FA, first author; NR, not reported.

Nine articles that collectively included 418 cases and 438 controls were used to evaluate the relationship between Oct-4 and cancer stage. When all eligible studies were pooled in the meta-analysis, there was evidence of an association between positive/high Oct-4 and cancer stage III/IV in different cancers. As show in Figure 2B, significant main effects were observed between Oct-4 and cancer stage III/IV (positive/high Oct-4 versus negative/low Oct-4: OR = 1.53, 95% CI = 1.12 – 2.10, *P* = 0.008).

**Figure 3:**
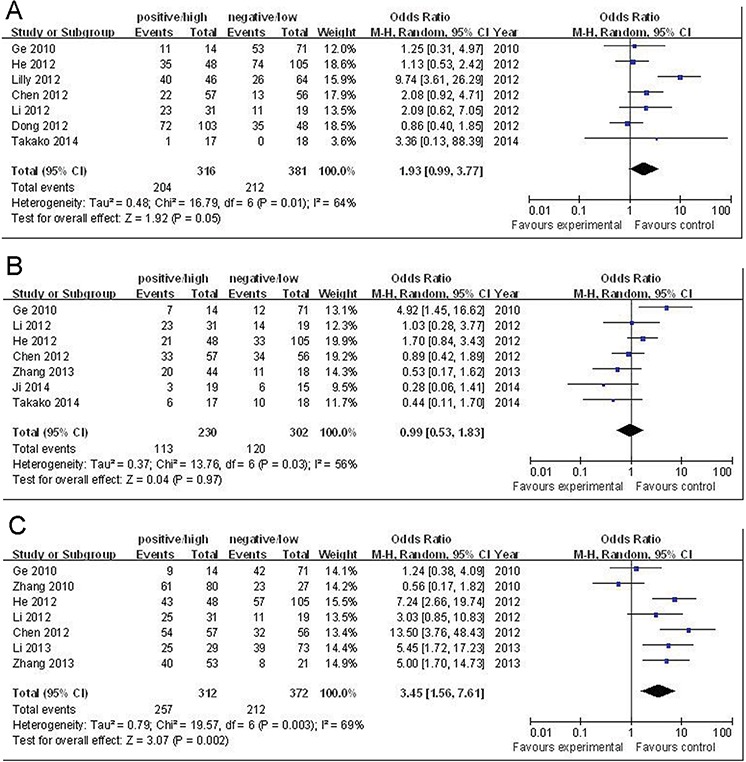
**A.** Forest plots of Oct-4 and primary tumor (T3-4) in all cases (positive/high Oct-4 vs. negative/low Oct-4). **B.** Forest plots of Oct-4 and lymph nodes (N1-2) in all cases (positive/high Oct-4 vs. negative/low Oct-4). **C.** Forest plots of Oct-4 and cancer grade of differentiation (intermediate-poor) in all cases (positive/high Oct-4 vs. negative/low Oct-4). The squares and horizontal lines correspond to the study-specific OR and 95% CI. The area of the squares reflects the weight (inverse of the variance). The diamond represents the summary OR and 95% CI.

Seven articles that collectively included 316 cases and 381 controls were used to evaluate the relationship between Oct-4 and primary tumor. As shown in Figure 3A, an analysis stratified by primary tumor (T_3–4_) showed that a significant main effect remained (positive/high Oct-4 versus negative/low Oct-4: OR = 1.93, 95% CI = 0.99 – 3.77, *P* = 0.05). However, as shown in Figure 3B, an analysis stratified by lymph nodes (N_1–2_) showed that there was no significant main effect remained (positive/high Oct-4 versus negative/low Oct-4: OR = 0.99, 95% CI = 0.53 – 1.83, *P* = 0.97).

Seven articles that collectively included 312 cases and 372 controls were used to evaluate the relationship between Oct-4 and cancer grade of differentiation (intermediate-poor). As show in Figure 3C, an analysis stratified by cancer grade of differentiation showed that the main effect remained (positive/high Oct-4 versus negative/low Oct-4: OR = 3.45, 95% CI = 1.56 – 7.61, *P* = 0.002).

### Tests of heterogeneity

Statistically significant heterogeneity was observed between trials of the following analyses using the Q statistic: Oct-4 with cancer stage III/IV, positive/high Oct-4 versus negative/low Oct-4 (*P* = 0.19, *I^2^* = 29%); Oct-4 with primary tumor (T_3–4_), positive/high Oct-4 versus negative/low Oct-4 (*P* = 0.01, *I^2^* = 64%); Oct-4 with cancer grade of differentiation, positive/high Oct-4 versus negative/low Oct-4 (*P* = 0.003, *I^2^* = 69%). There was no obvious heterogeneity in Oct-4 versus cancer stage; thus, the analysis was performed using a fixed-effects model. For primary tumor and cancer grade of differentiation, which exhibited moderate heterogeneity, analyses were performed using a random-effects model.

## DISCUSSION

TNM staging is determined based on the outcomes of physical examination, biopsy, and imaging tests. [31, 34]. In clinical application, a pathology report forms the basis of TNM staging. In addition to anatomic tumor categories, most sites and tumor types have a TNM staging system, [31, 32] with the anatomic extent of the tumor determining the cancer stage. Predicting survival probability and distinguishing among stages requires that each stage be homogeneous. Stages I to III correspond to progressively higher mortality due to localized and regional cancer. Stage IV is equivalent to systemic metastases. An accurate staging system is crucial for obtaining prognoses and guiding physicians in treatment strategies. [35]

Oct-4 is an important transcription factor that contributes on pluripotent stem cells. [36] Reports have shown that sustained expression of Oct-4 in epithelial tissues leads to dysplastic changes through inhibition of cellular differentiation; these actions, which are similar to the effects of Oct-4 in some progenitor cells, suggest that Oct-4 may serve the genesis of tumors. [37] However, the mechanisms by which Oct-4 acts during tumor progression, and notably the relationship between Oct-4 and cancer stage, have remained poorly understood.

Several clinical studies, including those by Ge et al., [22] Dong et al., [23] He et al., [25] Li et al., [27] and Ji et al. [29], have reported that positive/high Oct-4 s strongly and significantly associated with cancer stage. And it has been found that the expression of Oct-4 in malignant pleural effusion significantly related to distant metastasis and stage, as well as inversely correlated with patient survival. [38] The aim of the current study was to elucidate the relationship between Oct-4 and cancer stage. Our meta-analysis provided evidence of a significant association between positive/high Oct-4 and cancer stage III/IV in different cancers. In addition, analyses stratified by primary tumor (T_3–4_) and cancer grade of differentiation (intermediate-poor) showed a significant relationship with Oct-4.

The relationship of Oct-4 with cancer staging parameters suggests that positive/high Oct-4 can further inform the judgment of cancer malignancy based on basic TNM staging. Further large-scale investigations of Oct-4 and cancer stage are needed to confirm our results, but as additional evidence accumulates, Oct-4 could prove to be an important biomarker for cancer malignancy. More importantly, in a total of 11 articles on Oct-4 and cancer staging, there was only one paper related to analyzing the data in terms of Caucasian and the rest of the papers were Asian-related articles. The bewilderment that whether there were any possible bias related to racial issue seemed to need more time to consider.

More importantly, several Oct-4 pseudogenes were recently reported to be transcribed in cancer cells. [39–43] For example, POU5F1B, a processed pseudogene that was highly homologous to Oct-4, was conferred an aggressive phenotype on gastric cancer and associated with poor prognosis. [39] OCT4-pg4, another pseudogene of OCT4, was found abnormally activation in hepatocellular carcinoma. [41] To date, by using RT-PCR and sequencing analysis, three Oct-4 pseudogenes, including OCT4-pg1, OCT4-pg3, and OCT4-pg4, were found to be expressed in some solid tumors, glioma, and breast carcer, from which cancer stem cells had earlier been isolated. [39–43] Interestingly, controversy existed that pluripotency-associated isoform Oct4A was not expressed by malignant human urothelium, [40] and it was shown that some Oct-4 pseudogenes seemed be short of Oct-4 activities. Thus, whether Oct-4 expression in several cancer cells could actually be attributed to Oct-4 pseudogene expression remained unclear.

Some limitations of this meta-analysis should be noted. First, it was based on pooled positive or high Oct-4 data; thus, we cannot separately assess the relationship of Oct-4 positivity and high-level expression of Oct-4 with cancer staging parameters. Second, the small-study effect, in which effects reported in small studies are larger, could not be avoided because in some cases the study population was relatively small (< 500). The small number of cases included in order to assess the relation of Oct-4 to other clinicopathologic parameters may set a limit to the conclusions drawn. As noted above, further large-scale studies with more detailed individual data are warranted to further validate the relationship between Oct-4 and cancer stage. Experiments as to the biological role of Oct-4 in cancer progression and possibly a statistical analysis of a larger data set including an analysis of the types of cancers associated with Oct-4 expression would be helpful.

## MATERIALS AND METHODS

### Publication search

Computer searches were carried out using the following databases: PubMed, ISI Web of Knowledge, EMBASE, Google Scholar, China National Knowledge Infrastructure (CNKI), Chinese BioMedical Literature Database (CBM), and Wanfang database in China. The keywords were Oct-4, malignancy, neoplasm, cancer staging, and TNM staging. Articles published as of May 2015 that included case-control or cohort studies related to the association of positive/high Oct-4 with cancer stage were collected.

### Inclusion criteria

The following criteria were used to select publications for further meta-analysis: (1) published in English and Chinese regardless of publication time; (2) evaluated associations between positive/high Oct-4 and cancer stage; (3) confirmed cancer patients pathologically; (4) included detailed cancer/TNM staging data; and (5) compared at least two groups (i.e., positive Oct-4 vs. negative Oct-4 or high Oct-4 vs. low Oct-4).

### Data collection

Publication characteristic details, such as first author's name, publication year, patients' country of origin and ethnicity, total number of patients, cancer type, middle/mean age of the study population and disease stage, were collected for each eligible publication. Positive or high Oct-4 and cancer/TNM stage was end points of interest.

### Statistical analysis

Patients were divided into positive/high Oct-4 and negative/low Oct-4 groups. The associations between positive/high Oct-4 and cancer stage were determined by measuring odds ratio (OR) and associated 95% confidence intervals (CIs). The significance of the pooled OR was determined by the Z test. Statistical heterogeneity among studies was assessed with Cochran's heterogeneity statistic, Q, and *I^2^*, the latter of which describes variation that is due to heterogeneity rather than random error, as follows: *I^2^* = 0 – 25%, no heterogeneity; *I^2^* = 25 – 50%, moderate heterogeneity; *I^2^* = 50 – 75%, large heterogeneity; *I^2^* = 75 – 100%, extreme heterogeneity. [33] A fixed-effects model was applied in the initial analysis, and if significant heterogeneity existed, a confirmed random-effects model was used. Publication bias was evaluated using a funnel plot. All statistical analyses were carried out using Review Manager version 5.0 (Revman; The Cochrane Collaboration, Oxford, UK). All *P*-values in the meta-analysis were two-sided, and a *P*-value less than 0.05 was considered significant.

## CONCLUSION

In summary, our meta-analysis provides evidence of an association between positive/high Oct-4 and cancer stage, especially in Asian. Thus, positive/high Oct-4 may play a role in contributing to higher-grade cancer. Further studies based on various cancer types are warranted to verify our findings.
